# Farmers’ perceptions of climate variability and adaptation strategies in the rural areas of Dire Dawa administration, eastern Ethiopia

**DOI:** 10.1016/j.heliyon.2023.e15868

**Published:** 2023-05-11

**Authors:** Ahmed Jibril Usmail, Mengistu Mengesha Maja, Abebe Aschalew Lakew

**Affiliations:** a College of Natural Resources and Environmental Sciences, Oda Bultum University, Ethiopia; b College of Agriculture and Environmental Sciences, Haramaya University, Ethiopia; c Department of Natural Resources Management, Bahir Dar University, P.O.Box 79, Bahir Dar, Ethiopia

**Keywords:** Adaptation strategy, Rainfall variability, Temperature variability, Rainfall trends, Length of growing period, Onset of rainy season, Rainfall anomaly, Perceptions

## Abstract

Climate variability has significant impact on agricultural production especially in low-income countries where agriculture largely relies on rainfall, but only a few studies explored this issue at local scale. Therefore, this study was conducted to characterize local climate and assess farmers' perceptions and adaptation strategies to climate variability in the rural areas of Dire Dawa administration. Historical rainfall and temperature data (1987–2017) were obtained from National Meteorological Agency (NMA) of Ethiopia, while data of farmers' perceptions and adaptation strategies were collected from a total of 120 household heads through survey questionnaire, key informant interviews and focus group discussions. The results revealed that the area received an average annual rainfall of 568.3 mm with main rainy season (*kiremt*) contributing 70.7% to annual rainfall. The earliest and latest onset dates of *kiremt* season were 15^th^ of April and 2^nd^ of August, respectively. The amount of annual and *kiremt* rainfall totals showed low and medium variability with a coefficient variability (CV) of 18.3% and 27.7%, respectively, whereas short rainy season (*belg*) rainfall had high variability with a CV of 43.9%. Climate variability perception analysis showed that an overwhelming majority of the respondents (90%) perceived a decrease in the annual rainfall and 91.7% detected an increase in annual average temperature in the study area. Farmers of the study area were well aware of the changes in rainfall and temperature and thus employed a range of adaptation practices. Soil and water conservation practices (100%), off-farm income diversification (63%), planting drought-tolerant varieties (50%) and changing of planting date (45%) were the main adaptation strategies employed in the study area to avert the negative effects of climate variability. The findings imply that the area has been experiencing palpable changes in climate variables during the study period against which farmers exercised multiple adaptation strategies. However, farmers in the area are still face hardship as a result of climate variability which necessitates improving farmers’ resilience through innovative mechanisms and better extension services.

## 1 Introduction

Climate variability is among the major challenges facing agricultural productivity in twenty-first century. It is intricately linked to agricultural production and causes significant loss of agricultural yield in developing countries with rainfall dependent agriculture and limited adaptation capacity [1,2]. The negative impact of rising temperature and frequent droughts on agricultural productivity is substantial in areas where crops are highly dependent on rainfall, jeopardizing food security and socioeconomic development [3]. Drought-induced food insecurity has been one of the greatest challenges of countries where agriculture is largely dependent on rainfall [4,5].

Arid and semi-arid regions make up more than 75% of Ethiopia's land mass [6], lack sufficient rainfall to support agricultural activities [7]. Agricultural sector accounts for 33.88% of the country's gross domestic product, employs 79% of the population, and accounts for 79% of the country's foreign earnings [8]. This sector has been directly threatened by rising temperatures and extreme rainfall variability [[9], [10], [11]]. Previous studies [eg. [[12], [13], [14], [15]]] reported erratic rainfall, prolonged drought, and rising mean temperature in many parts of Ethiopia, resulting in lower crop production, fertilizer use and gross domestic product at the local, regional and national levels. In addition to crop and livestock production, climate variability also affects the environment, water resources, human health and farmers' livelihoods [16,17]. The adverse impact on agricultural production is likely to worsen in Ethiopia as recent climate projections for the country suggest a rise in temperature, while annual and *belg* (short rainy season from February to April) seasonal rainfall are expected to decline in the next decades [18].

Climate variability and change are major threats to the livelihood of smallholder farmers in the eastern part of Ethiopia, including Dire Dawa administration and its surrounding communities [19,20]. As a result of increasing population, agricultural land expansion, resettlement, land degradation and climate change, the overall natural resource base of Dire Dawa administration has been severely degraded. Mild to severe droughts, flash floods and land degradation put the livelihood and food security of smallholder farmers at risk in Dire Dawa administration and surrounding communities [21]. Effective implementation of adaptation measures against the impacts of climate variability and change requires farmers' knowledge and perceptions of the changes [[22], [23], [24], [25], [26]]. Furthermore, farmers' knowledge and perceptions of climate variability and change, and adaptation to its adverse impacts may differ depending on the local climate regime, the magnitude of impact the change, and other socioeconomic factors [27]. Previous assessments of climate variability, farmers' perceptions of the changes and adaptation strategies are usually carried out at country or regional scale in Ethiopia [28,29]. District level studies that capture local changes better were also conducted in some parts of the country to assess location-specific climate risks and adaptation practices exercised by farmers [15,30]. These studies showed location-specific variations in rainfall and temperature features; socioeconomically and agro-ecologically different perceptions of climate variability as well as variations in adaptation responses to climate extremes that were location- and context-dependent [26,[31], [32], [33]]. Conducting climate variability analysis and assessing its impacts at local level is helpful to better comprehend farmers’ perceptions [34] and plan adaptation strategies that better fit the locality in a participatory approach.

Accumulating evidence indicates low to extreme variation in rainfall and rising temperature in many parts of Ethiopia [ [15,18,19]]. Delayed onset of main rainy season, extended dry spell, early cessation as well as decreasing amount of *belg* rainfall are among the changes noted by previous studies [19,35]. In recent years, Dire Dawa administration has been affected by climate extremes especially occasional heavy rainfall in the surrounding areas causing flash floods that led to death, displacement of human beings and destruction of properties in the administration. On the other hand, decreasing and erratic rainfall posed threat to agricultural activities and livelihoods of smallholder farmers in the area. Although there are studies that explored climate variability in different parts of the country, no sufficient research documented climate variability, farmers' perceptions and adaptation strategies in the rural areas of Dire Dawa administration. Therefore, this study aims to fill this information lacuna about climate variability, farmers' perception of the variability, and adaptation strategies exercised by farmers in the study area. Assessing farmers' perceptions of different aspects of climate variability and change is essential to devise more effective, location-specific adaptation strategies. Moreover, incorporating the experiences of farmers regarding changes in climate variability in their locality could offer important insights that might not be captured by the analysis of recorded data alone. Thus, scientific information of climate variability and change supported by local knowledge has paramount importance to advance understanding and identifying new adaptation strategies that better fit the local condition. Therefore, the objective of this study was to assess climate variability, farmers’ perceptions of the variability and adaptation strategies practiced against the impacts of climate variability in the rural areas of the Dire Dawa administration.

## 2 Materials and methods

### 2.1 Description of the study area

**Fig. 1:**
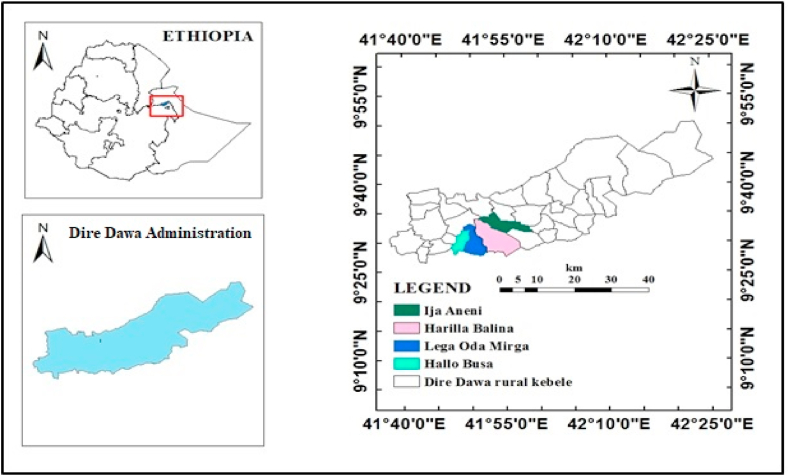
Map of the study area.

This study was conducted in the rural areas of Dire Dawa administration of Eastern Ethiopia (Fig. 1). It is located at a distance of 515 km southeast of Addis Ababa, the capital city of Ethiopia, and 45 km from Harar town, UNESCO world Heritage site. Geographically, the study area extends from 9° 27′ to 9° 49′ latitude and 41° 38′ to 42° 19′ longitude. The study area is bordered by Somali national regional state in the west and Oromia national regional state in the north, south and east. The administration has 10 urban and 38 rural *kebeles* (smallest administrative unit in the Federal Democratic Republic of Ethiopia) with an approximate population of about 400,000 [36] and covers a total area of 133,262 ha, of which, 2.27% is urban and 97.73% is rural [37].

The topography of the landscape of the rural areas of DDA and its neighboring rural *kebeles* (sub districts) ranges from steep mountains to flat plains, with elevations varying from 950 to over 2000 m above sea level [36]. Flash floods caused death and destruction in the study region due to the steep slope in the surrounding area and heavy rainfall over short period of time [21]. The area is classified as a hot semi-arid zone agro-ecologically, with an average annual maximum temperature of 32.1 °C and a mean annual minimum temperature of 19.1 °C. The area experiences bimodal rainfall, with the short rainy season (*belg*) lasting from January to March and the long rainy season (*kiremt*) extends from June to August [38]. The area receives the average annual rainfall of 638 mm, with most of the rain falling during the extended rainy season. Fluvisols are the most common soil types with topography, parent material, and slope all having an impact on soil formation. Mixed farming system that involves both crop production and livestock rearing accounts for 93% of the households’ livelihood in the study area [39].

### 2.2 Sampling procedures and sample size

Purposive and simple random sampling techniques were used to select the *kebeles* and households. First, four rural *kebeles* were purposively selected from the 38 rural *kebeles* considering susceptibility of the area to climate extremes related stresses such as erratic rainfall, frequent drought, crop pests, and livestock diseases as well as highly undulating topography amongst others. Accordingly, Harilla consisting of 1095 households, Hallo Busa (986 households), Lega Oda Mirga (1765 households) and Ija Aneni consisting of 723 (households) were considered for the survey. One hundred twenty households were selected by simple random sampling from a total population of 4569 households using a household list provided by *kebele* administrations*.* Simplified formula (equation (1)) provided by Ref. [40] was used to determine the required sample size at 95% confidence level, 5% degree of variability and 9% level of precision.

Equation 1 $$n=\frac{N}{1+{Ne}^{2}}$$

where, n is sample size, N is the number of farmers in the selected *kebeles* and e is the desired level of precision.

### 2.3 Data source and methods of data collection

Both primary and secondary data sources were used to collect data to deal with the research objective. Primary data were collected through survey questionnaire, and supplemented by key informant interviews, focus group discussions, and direct observations in the field.

The household survey questionnaire was the main data collection method used in this study. Open and close-ended questions were prepared to acquire information regarding socioeconomic characteristics, farming features, livelihood activities, and farmers’ perceptions of climate variability as well as adaptation practices. The questions were prepared in English language and translated to local language (*Afaan Oromo*) and distributed to respondents by enumerators. The survey respondent in each household was the head or partner in the absence of household head. Face-to-face interviews were conducted in their homes or on the farms of respondents where appropriate.

A focus group discussion, frequently used as a qualitative approach to gain an in-depth understanding of socioeconomic issues, was used to supplement data acquired through household survey. A total of four focus group discussions, each comprising a minimum of six purposively selected individuals were conducted in the study area. For this, open-ended questions were prepared to gather qualitative data concerning climate variability, farmers’ perceptions of the changes and adaptation practices frequently used in the study area [41].

Furthermore, key informant interviews were performed with knowledgeable individuals from each *kebeles*. To get detailed information about the issues pertinent to the research objective, check list was prepared and interviews were conducted with individuals that were purposively selected. The interviews were conducted in each selected *kebeles* with development agents, local leaders, extension workers and agricultural office representative in the study area [42]. In addition to the above data collection methods, personal observation was conducted in the field in the study area, while also interacting with farmers in the field.

Secondary data were obtained from National Meteorological Agency (NMA) of Ethiopia. Historical rainfall (mm) and temperature (^o^C) (1987–2017) data of the study location were acquired from NMA of Ethiopia. Data was entered using the days of year (DOY) entry format into a Microsoft Excel 2013 spreadsheet.

**Ethical issues:** The Review Board at Oda Bultum University had provided all ethical approval before the research was carried out. The authors confirm that informed consent was obtained from all participants. Keenly aware of ethical issue, the authors adhered to key principles of research ethics including the purpose of the research to participants, seeking and obtaining informed consent, ensuring voluntary participation and confidentiality of participants. The participants were also informed about the anonymity of the data collected and these data would be used for scientific research only.

### 2.4 Data quality control

Prior to analysis, the observed climatic data were plotted against time and visually inspected for discontinuities as well as special codes for missing values. Special codes were eliminated, and typing errors were handled on a case-by-case basis, based on data from the day before and after the occurrence, as well as information from surrounding stations.

### 2.5 Data analyses

Rainfall and temperature data were analyzed using variability and trend analysis. As statistical descriptors of rainfall variability, coefficient of variation (CV) and percentage deviation from the mean (anomalies) were used. Standard rainfall anomalies were plotted against time (in years) to visualize the time-series variation of annual and seasonal rainfall with the mean. The Standardized Rainfall Anomaly (SRA) was calculated as the difference between the annual/seasonal totals of a particular year and the long term average rainfall records divided by the standard deviation of the long term data [43,44]. Rainfall anomaly was used to examine the nature of rainfall over the period of observation and to determine dry and wet years in the record [45]. Mathematically it is computed as in equation (2):

equation 2 Z=(x−μ)/δ

where, Z is standardized rainfall anomaly; X is the annual/seasonal rainfall total of a particular year; μ is mean annual/seasonal rainfall over a period of observation and ***δ*** is the standard deviation of annual/seasonal rainfall over the period of observation. Based on Z values, drought severity classes are given as extreme drought (*Z* < −1.65), severe drought (−1.28 >*Z* > −1.65), moderate drought (−0.84 >*Z* > - 1.28) and no drought (*Z* > −0.84) [46].

Rainfall and temperature trends were analyzed using Mann-Kendall (MK) test, a widely used non-parametric method, which is appropriate for data with outliers and not normally distributed [47]. The MK test was employed in this study to determine whether monthly, seasonal, and annual rainfall and temperature had increasing or decreasing trend in the study area. The Mann-Kendall test statistic is calculated as follows (equation (3)):

equation 3 $$S=\sum_{i=1}^{N-1}\sum_{j=i+1}^{N}sgn\left(Xj-Xi\right)$$

where, S is the Mann-Kendall's statistic; xi and xj are the time series' sequential data values in the years I and j (j > I and N is the time series' length. A positive S value shows an increasing trend in the data series, while a negative value indicates a decreasing trend. The sign function is given in equation (4):

equation 4 $$sgn\left(x_{j}-x_{i}\right)=\left\{ \begin{array}{c} +1\;if\;\left(x_{j}-x_{i}\right)>0\\ 0\;if\;\left(x_{j}-x_{i}\right)=0\\ -1\;{if\;(x}_{j}-x_{i})<0 \end{array}\right.$$

The variance of *S*, for the situation where there may be ties (that is equal values) in the *x* values is given in equation (5):

equation 5 $$var\left(s\right)=\frac{1}{18}\left[N\left(N-1\right)2N+5)-\sum_{i=1}^{m}t_{i}\left(t_{i}-1\right)(2t_{i}+5\right]$$

where, *m* is the number of tied groups in the data set and *ti* is the number of data points in the *i*th tied group. For n larger than 10, Z_MK_ approximates the standard normal distribution [48] and computed as follows in equation (6):

equation 6 $$Z_{MK}=\left\{ \begin{array}{c} \frac{s-1}{\sqrt{var\left(s\right)}}\;if\;s>0\\ 0\;if\;s=0\\ \frac{s+1}{\sqrt{var\left(s\right)}}\;if\;s<0 \end{array}\right.$$

The presence of a statistically significant trend is evaluated using the Z_MK_ value. In a two-sided test for trend, the null hypothesis, Ho should be accepted if |ZMK|<Z1−∂2 , at a given level of significance. Z1-α/2 is the critical value of ZMK from the standard normal table. This test is applied in cases where the trend is assumed to be linear, depicting the quantification of changes per unit time. Based on the earlier procedure trends of annual, seasonal (*kiremt* and *belg*) and monthly (January to December) totals were analyzed. Thus, the magnitude is usually determined by Sen's test [49] which is also a nonparametric technique and calculated as in equation (7):

equation 7 $$T_{i}=\frac{X_{i}-X_{k}}{J-K}$$

where, i = 1, 2, 3 … N, xj and xk represent data values at time *j* and *k*, respectively.

equation 8 $$Qi=\left\{ \begin{array}{c} T\frac{T+1}{2}\;N\;is\;odd\\ \frac{1}{2}\left(T_{\frac{N}{2}}+T_{\frac{N+2}{2}}\right)\;N\;is\;even \end{array}\right.$$

A positive *Q*i value represents an increasing trend; a negative *Qi* value represents a decreasing trend over time. Mann-Kendall test with Sen's slope estimator was applied for nonparametric test and simple linear regression for parametric test (equation (8)).

Smallholder farmers' perceptions of change in various climatic variables over the last 30 years were also collected using a 5-point Likert scaled attitude items: strongly disagree, disagree, neutral, agree and strongly agree [50]. In the case of adaptation strategies, the respondents were asked about their use of adaptation practices. Descriptive statistical tools such as, mean, percentages, frequencies as well as Likert rating scale were used to characterize farmer's perceptions on climate variability and changes and various adaptation measures being used by farmers. Farmers who strongly agree and who simply agree were considered perceived the climate variability and change.

Lack of complete set of climate data in the study area for a study period longer than indicated in this study was the main limitation of the study. A combination of various challenges compelled the authors to limit survey to only 120 households.

## 3 Results and discussion

### 3.1 Annual, seasonal and monthly rainfall variability

The mean annual rainfall of Dire Dawa administration over the study period was 568.3 mm, with a CV of 18.3% and an SD of 104 mm (Table 1). The annual mean rainfall amount is comparable to the amount (634 mm) reported for Dire Dawa administration by Ref. [21]. The CV value indicates that there was a moderate inter-annual fluctuation during the study period. Similar studies in eastern Ethiopia also reported fluctuation of monthly, seasonal and annual rainfall. For instance Refs. [39,51], reported changes in rainfall and temperature in East Hararghe zone, an adjacent area to Dire Dawa administration in recent decades. Climate studies in different parts of the country indicated variability in rainfall although the magnitude and trend of the variability differed across location and time period [15,26,45,52]. However, since Dire Dawa administration is mainly lowland and dry land area, mean annual rainfall amount recorded was lower than the rainfall amount observed in southern, northern and central regions of Ethiopia [[52], [53], [54]]. The mean maximum and minimum recorded rainfalls were 367.8 mm and 753 mm, respectively.

**Table 1:** Descriptive statistics of monthly, *belg, kiremt* and annual rainfall in Dire Dawa administration.

	Descriptive Statistics of rainfall				
	Min	Max	Mean	SD	CV%
January	0	39	12.4	14.6	118
February	0	55	15.38	18.9	123
March	0.2	72.4	71.57	51.6	72.1
April	1.8	107.8	92	58.4	63.6
May	0	132.4	48.7	34.9	71.6
June	0.2	30	14.5	8.3	57.3
July	46.9	136.3	89.6	25	27.9
August	53.1	180	113.3	34.29	30.3
September	16.2	152.2	71.1	34.6	48.9
October	0	85.6	23	24.3	105.5
November	0	82.1	14.1	20.75	146.8
December	0	71.7	9.59	18.1	188.6
*Kiremt*	145	466.6	303.3	83.9	27.7
*Belg*	47.4	445	241.3	106.3	43.9
Annual	367.8	753	568.3	104.3	18.3

Note: min, minimum; max, maximum; SD, standard deviation; CV, coefficient of variation.

The mean maximum and minimum recorded rainfalls were 367.8 mm and 753 mm, respectively. The mean *belg* rainfall was 243.1 mm with a CV of 43.9%, and an SD of 106.3 mm in the study area. This implies that rainfall during *belg* season has been unreliable and difficult to forecast in recent decades, potentially limiting pasture production and water supply for livestock. Similarly, *belg* rainfall was reported to have shown considerable variation in different parts of the country compared to *kiremt* and yearly total rainfall during different time patches [10,44]. Regarding the amount of *belg* rainfall, similar studies in various parts of the country also revealed a decreasing trend and substantial variation in *belg* rainfall [19,26,45]. In terms of agriculture, frequent failure and lack of consistency in *belg* rainfall impedes harvest during small rainy season and threatens the area's food security status [55]. *Belg* rainfall is also a lifeline for livestock as it recharges surface and ground water which serve as water source for domestic animals [56].

The mean *kiremt* seasonal rainfall was 303.3 mm, with a moderate CV of 27.7% and an SD of 83.9 mm (Table 1). The CV value indicates that *kiremt* seasonal rainfall was less variable than *belg* rainfall, which had a CV of 43.9%. In most parts of the country, *belg* rainfall is highly unreliable and erratic. For example [45], reported more variability in *belg* rainfall than the *kiremt* rainfall in central Ethiopia. Seleshi and Zanke [57] also discovered considerable variability (CV>30%) in *kiremt* rainfall over the eastern and southern stations of Dire Dawa, Jijiga, and Negele. In general, the *kiremt* months (June, July, August and September) deliver a significant quantity of rainfall, accounting for over 70% of total annual rainfall in the study area, which is similar to the proportion of *kiremt* rainfall contribution (74.4%) to the annual rainfall in central Ethiopia [45]. Farmers in Ethiopia are highly dependent on *kiremt* seasonal rainfall for crop production [58], and even little variations in amount, intensity, length, onset, and cessation have a direct impact on agricultural production.

### 3.2 Onset and end date of rainy season

As indicated in Table 2, the mean onset date of *kiremt* seasonal rainfall was June 24 (176 DOY) with a CV of 19.8% and an SD of 34.7 days. The *kiremt* rainy season started on the 15th of April (106 DOY) and ended on the 2nd of August (215 DOY). In the study area, the rainy season used to start in the first week of April, but now it comes mid-April (a two-week delay), forcing farmers to change their agricultural activities such as land preparation and sowing dates. Rainy season start dates were more variable than end dates, requiring careful management of agricultural operations. Similar variations in rainfall events such as onset date, end date, growing period, and dry spell length were observed in northern Ethiopia [44]. In Northwestern Ethiopia [45], found a shift in the agricultural calendar due to changes in onset and end dates. However [59], found no significant variation in onset, cessation, or length of the growing period in the Baro-Akobo river basin in the last three decades of 20th century. This variation of onset and end dates of rainfall is consistent with past and projected changes in rainfall features for east Africa in general and Ethiopia in particular [18,60]. Extreme variation of onset and end dates of rainfall in some places of lack of palpable changes in the rainfall features suggest that climate variables differ across locations.

**Table 2:** Descriptive statistics of onset date, end date, length of growing period in Dire Dawa administration.

Descriptive statistics	Rainfall features		
	SOS (DOY)	EOS (DOY)	LGP (DAYS)
Min.	106	302	95
Max.	215	363	238
Mean	176	319	143
SD	34.7	17.3	41.0
Trend	0.18	−0.01	−0.13
Slope	0.52	0.39	−0.80
Q1 (25%)	166	305	110
Median (50%)	189	316	131
Q3 (75%)	201	326	169
CV%	19.8	5.6	27.1

Note: Min, minimum; Max, maximum; CV, coefficient of variation; SD, standard deviation.

### 3.3 Length of growing period (LGP)

Climate data analysis revealed that the LGP ranged from 95 to 238 days, with a CV of 27.1%, (Table 2). The LGP in this study is consistent with the LGP (76–239 days) reported by Ref. [54] in central Rift Valley of Ethiopia. The moderate variation in the LGP observed necessitates a cautious approach by farmers with regards to planning agricultural activities in the study area. Farmers in arid and semi-arid areas require more accurate climate information to adjust agricultural production activities to delayed onset and/or early cessation of rainfall [61,62]. Shorter growing seasons and other climate extremes are one of the key causes of agricultural failure in several dry land parts of Ethiopia [54,63].

### 3.4 Rainfall trend analysis

Table 3 shows the observed trends in yearly and seasonal rainfall totals. Annual, *belg* and *kiremt* rainfall revealed a statistically non-significant declining trend by factors of 0.12, 0.4, and 0.16 mm per year, respectively. Previous studies of rainfall trend analysis in Ethiopia had mixed findings with some reporting upward trends whereas others reporting downward trends in different parts of the country [15,45,64]. In Woleka sub-basin of north central Ethiopia [45], reported a mixed trend, with different months showing statistically significant and non-significant decreasing trends. Eshetu et al. [65] found non-significant annual and seasonal rainfall trend in southwestern Ethiopia, while [66] showed positive trend in seven and negative rainfall trend in four meteorological stations in central Ethiopia. The wide variation in rainfall indicates the presence of multiple factors that could influence climate which might be associated to diverse physiography and agro-climatic zones across the country.

**Table 3:** Mann-Kendall trend test statistics result for annual, monthly and seasonal rainfall at Dire Dawa administration.

Climatic variables	MK	Sen's Slope
January	−0.19	0.00
February	−2.26*	−0.26
March	−0.12	−0.92
April	−0.27	−2.44
May	0.10	0.31
June	−0.05	−0.01
July	0.09	0.45
August	0.17	1.10
September	0.12	0.77
October	−0.13	−0.26
November	0.16	0.00
December	−0.20	0.00
*Kiremt*	−0.16	−3.10
*Belg*	−0.40	−6.32
Annual	−0.12	−2.713

Note: MK: Mann-Kendall, *: statistically significant at *P* < 0.05.

### 3.5 Analysis of rainfall anomaly

**Fig. 2:**
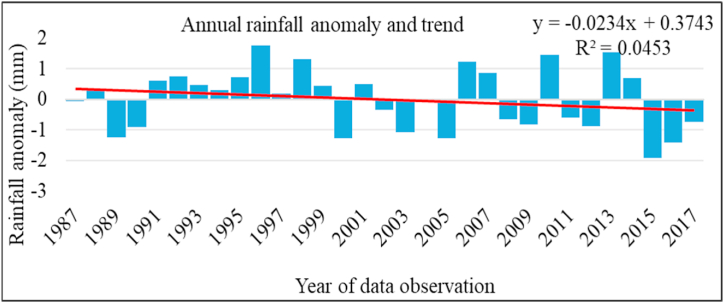
Annual rainfall trend and a standardized anomaly.

The study area experienced alternating dry and wet patches over the study period (Fig. 2). Of the observed 31 years, 14 years (45.2%) had below long-term average and 17 years (54.8%) had above long-term average annual rainfall. Most of the negative anomalies occurred during early 2000s and, during and after 2015. The negative anomaly years from 2015 to 2017 are linked with failure in rainfall as a result of El-Niño phenomenon in the Horn of Africa [67]. The same authors demonstrated that up to 50% of rainfall anomalies during long rainy season in Ethiopia were driven by variation in equatorial sea surface temperature. Other studies [e.g. 45,68,69] also showed negative rainfall anomalies in various parts of Ethiopia over different time periods. Inter-annual variability and drought years have become more frequent in the last five decades.

### 3.6 Temperature variability and trend

The variability of monthly, seasonal and annual maximum temperature is presented in Table 4. The annual maximum temperature varied between 31 and 34 °C with an average value of 32.4 °C in Dire Dawa administration during the study period. The Mann-Kendall trend test showed that maximum and minimum annual temperature showed increasing trend by a factor of 0.74 °C and 0.15 °C per year, respectively. A number of studies explored temperature in various parts of Ethiopia during the last few decades and reported warming trend in both minimum and maximum temperatures [45,58,69]. World Bank group [70] reported that the mean annual temperature increased by 1.3 °C between 1960 and 2006, an average rate of 0.28 °C per decade in Ethiopia. Similarly, both maximum and minimum temperatures tended to increase at different parts of the country in the last three to four decades [15,18,71]. This increasing trend of already high temperature in Dire Dawa administration contributes to high evapotranspiration, which causes loss of soil moisture and leads to a reduction in agricultural production in the study area.

**Table 4:** Mann-Kendall statistics result for monthly, *belg, kiremt* and annual maximum temperature.

Climatic variables	Descriptive statistics				
	Min	Max	Mean	MK	Sen's slope
January	27	30	29	0.42	0.043
February	27	42	31	0.45	0.095
March	28	36	32	0.62	0.14
April	28	36	33	0.55	0.14
May	31	37	35	0.29	0.04
June	33	37	36	0.48	0.04
July	32	35	34	0.4	0.05
August	31	35	33	0.14	0
September	31	35	33	0.2	0
October	30	35	33	0.5	0.1
November	29	33	31	0.53	0.07
December	28	31	29	0.5	0.04
*Belg*	30	37	32.9	0.65	0.13
*Kiremt*	31	35	33.9	0.28	0.03
Annual	31	34	32.4	0.74	0.07

### 3.7 Farmers’ perceptions of climate variability and change

Farmers' perceptions of *belg*, *kiremt* and annual rainfall are presented in Table 5. The result showed that the majority of the respondents (93.3%) perceived at least an aspect of rainfall and temperature change in the study area during the last three decades. Accordingly, 90% of the sampled respondents perceived that annual rainfall has decreased, whereas only 6.7% perceived no change in the area over the study period. In terms of *belg* seasonal rainfall, about 85.8%, 8.3% and 6.7% of respondents perceived a decline, an increase and no change, respectively during the study years. Furthermore, about 74% of the respondents felt a decrease in *kiremt* seasonal rainfall, while 15.8% of the respondents perceived an increase in *kiremt* rainfall over the study period. The high proportion of respondents perceiving a decrease in *belg* rainfall is also reflected in the analyzed meteorological data, which showed a decreasing trend during the study years. The vast majority, 96% and 98.3% of the respondents perceived late onset and early cessation of rainfall. Farmers’ perceptions of climate variability may diverge from or contradict data from meteorological stations [15,72]. In this study however, perceptions of respondents are in conformity with results from analysis of meteorological data. Actual climate variability was not perceived equally by all smallholder farmers; for instance, farmers of arid and semi-arid lowlands perceive the changes in climate features better than farmers from highlands [30]. Lower proportion of respondents perceived changes in climate features in adjacent Hararghe highlands [26] compared with the lowland Dire Dada administration. The high perceptions of climate variability among the respondents in the study area may be due to rapid changes in biophysical conditions of already water-stressed arid and semi-arid environment. Furthermore, access to climate and market information, agro-ecology, education, agricultural input, and market access can influence perceptions level of smallholders [15].

**Table 5:** Farmers’ perceptions of rainfall variability in Dire Dawa administration.

Climatic variables	Perceived change					
	Increase		Decrease		No change	
	N	%	N	%	N	%
Annual rainfall	4	3.3	108	90	8	6.7
*Belg* seasonal rainfall	10	8.3	103	85.8	7	5.8
*Kiremt* seasonal rainfall	19	15.8	89	74.2	12	10

Over 91% of the respondents perceived an increase whereas only 5.8% of the respondents perceived a decrease in temperature (Table 6). Kahsay et al. [73] also reported over 90% of the respondents perceived changes in temperature in Tigray Region of Ethiopia. In terms of annual and seasonal temperatures, 87.5% of the respondents recognized increase in temperature whereas 7.5% perceived a decrease. This indicates that the respondents were aware of variation in climate variables in the study area. The survey result is also in line with historical temperature data (1987–2017) from the nearby observatory station, which revealed increasing trend for annual minimum, maximum and seasonal temperatures in the study area.

**Table 6:** Smallholder farmers’ perceptions of temperature variability.

Temperature change over 30 years	Perceived change					
	Increase		Decrease		No change	
	N	%	N	%	N	%
Average annual temperature	110	91.7	7	5.8	3	2.5
Annual and seasonal temperature	105	87.5	9	7.5	6	5

Focus group discussants and key informant interviewees had similar views of temperature in recent years. One discussant stated the following to describe the challenge,

“Working in the farm during the day especially close to midday has become uncomfortable due to extreme day temperature; we must start our farm work late afternoon which has impact on working time.”

Another elderly farmer from the area, describing his experience with high temperature, added that*, “the land is extremely hot; it is as if the creator threw hellfire up on us.*” These extreme conditions described by the native elderly farmers in the study area are also common experiences of farmers in other dry lowland parts of Ethiopia [30].

Of the respondents, 96.7% perceived a decline in frequency of cloudy days and high day time temperature in the study area (Table 7). The high perception of climate change/variability among the respondents might be associated with agroecological setting as the study area is often vulnerable to moisture stress; the farmers are familiar with climate extremes in their daily life. Consistent with the findings of this study, 78% of interviewed households perceived an increase in temperature, while 83% felt a decrease in rainfall in eastern part of Ethiopia where the current study was conducted [26]. Perceptions apparently vary depending of multiple socioeconomic and biophysical factors [74].

**Table 7:** Scale of farmers’ perceptions of climate variability and change (1987–2017).

Climate variability signal and pattern of change	Level of perceptions (n = 120)					
	1	2	3	4	5	Mean
Late onset of rainy season	0.8	1.6	1.7	16.7	79.2	4.7
Early cessation of rainy season	0	0	1.7	25	73.3	4.7
High day time temperature	0	0.8	2.5	19.2	77.5	4.7
Increase in the number of hot days/period	0	0.8	1.7	10	87.5	4.8
Frequency of cloudy days decreased	0	0.8	2.5	4.2	92.5	4.9
Increase in recurrence of droughts	0.8	4.2	9.2	12.5	73.3	4.5

Note: 1 = Strongly Disagree, 2 = Disagree, 3 = Neutral, 4 = Agree, and 5 = Strongly Agree.

### 3.8 Adaptation strategies of farmers to climate variability

Major adaptation practices frequented by the respondents in the study area were presented in Table 8. In the study area, six main adaptation strategies against the impacts of climate and variability have been identified. All of the farmers (100%) practiced soil and water conservation to reduce hazards of climate change and variability. Considering most of the households own farm with diversified topographic features, it is not surprising that the farmers used soil and water conservation. Since the study area is a flood prone area, soil and water conservation practices are preferable to conserve top soil [75]. Similar proportion of farmers implemented adaptation practices in the farmers to reduce the adverse impacts of climate variability in Eastern highlands of Ethiopia. Focus group discussants and key informants concurred that farmers in the study area were avid users of soil and water conservation practices along with other adaptation strategies such as tree planting, construction of check dams and moisture conservation practices. However, it is imperative to focus on female household heads also since they are more vulnerable to climate variability and their ability to adapt to climate variability is constrained by lack of information and resource constraints [20].

**Table 8:** Widely used adaptation strategies by households of the study area.

Adaptation method used	Frequency (N)	Percentage (%)
Soil and water conservation	120	100
Income diversification	76	63
Planting early maturing and drought tolerant variety	60	50
Changing planting date	54	45
Cultivation of valley bottom	30	25
Handcraft	13	10.8

In times of drought, majority of the household heads (63%) reported that they diversify their income sources. The household heads frequently engage in off-farm activities such as selling of honey or home made products like mattresses, fast food, beverages and ropes. During dry season, women engage in petty trade to gain additional income for the household while young household members earn by taking up labor jobs such as cobblestone road construction and extraction of sand for construction. This is congruent with results in various studies as smallholders usually engage in off-farm activities to complement their household income [22,28,31].

Early or late onset of rainfall is a major challenge for the farmers, thus selection of varieties that are more suitable and resilient to moisture stress is a common adaptation strategy. Accordingly, 50% of smallholder farmers plant early maturing and drought tolerant varieties, which is an essential strategy to reduce risk of total yield loss. Focus group discussants and key informant interviewees also stated the use of early maturing and drought resistant sorghum variety locally called *isabakko and dukun* (sorghum variety of short height)*,* beans and short-cycle vegetables such as onion and tomatoes. Farmers inhabiting drought affected areas plant drought tolerant crops and varieties to reduce yield loss [24]. Moreover, changing of planting date was another important adaptation practice used against adverse effects of climate variability and change in the study area. From the total respondents, 45% changed planting date as an adaptation strategy to reduce the adverse effect of changing onset and end date on their farm productivity. Farmers were adjusting the time of planting date based on their experience in farming practices. Farmers’ use of diverse adaptation strategies suggests that one strategy is not enough to fend off the adverse effect of climate variability and change on their livelihood. In this regard [28,76], also showed that growing different crop types, changing planting dates, planting trees, adoption of drought tolerant and early maturing crop varieties, soil and water conservation techniques or soil erosion prevention programs are among adaptation strategies used against climate vagaries by smallholder farmers in many parts of Ethiopia.

## 4 Conclusion

The results of this study showed that the mean annual rainfall had a downward trend, while onset and cessation dates shifted in the area over the study period. The short rainy season (*belg*) rain showed high variability, and had a declining trend over the study years. *Kiremt* rainfall accounted for the majority of annual rainfall, and it is a season main agricultural operations in Dire Dawa administration and its surroundings highland areas. The maximum and minimum temperatures showed increasing trend over the study area during the studied years. The overwhelming majority (95%) of the farmers perceived an aspect of rainfall or temperature changed in the last three decades, which was in agreement with recorded climate data. A range of adaptation strategies were exercised by the farmers but the most frequently used adaptation strategies include soil and water conservation, income diversification, early maturing and drought tolerant varieties and changing planting date.

The findings of the current study imply that Dire Dawa administration experiences climate variability and that is constraining agricultural activities and thereby adversely affecting the rural communities’ livelihoods. Despite high awareness of climate variability and use of a range of adaptation measures, the farmers still experience the adverse impacts of climate variability and change in the area. Therefore, all stakeholders should strive to identify more location-specific adaptation practices to enhance resilience of farmers against climate variability. Investment on off-farm income sources, improved access to drought resistant varieties of crops grown in the area, and encouraging implementation of soil and water conservation practices are essential to combat the impact of climate variability in the area. Further studies that assess climate variability and its effect on the livelihood and adaptation strategies in Dire Dawa administration and surrounding villages might help to provide a better picture of climate variability and its impacts in the area.

## Author contribution statement

Ahmed Jibril Usmail: Conceived and designed the study, performed the study, analyzed and interpreted the data and wrote the paper.

Mengistu Mengesha Maja: Conceived and designed the study, analyzed and interpreted the data and wrote the paper.

Abebe Aschale Lakew: Conceived and designed the study, analyzed and interpreted the data and wrote the paper.

## Funding statement

This research received no specific grant from any funding agency in the public, commercial, or not-for- profit sectors.

## Data availability statement

Data included in article/supplementary material/referenced in article.

## Additional information

No additional information is available for this paper.

## Declaration of competing interest

The authors declare no conflict of interest.
